# Diversity of* Leptospira* spp. in Rats and Environment from Urban Areas of Sarawak, Malaysia

**DOI:** 10.1155/2017/3760674

**Published:** 2017-02-28

**Authors:** Chai Fung Pui, Lesley Maurice Bilung, Kasing Apun, Lela Su'ut

**Affiliations:** ^1^Department of Molecular Biology, Faculty of Resource Science and Technology, Universiti Malaysia Sarawak, 94300 Kota Samarahan, Sarawak, Malaysia; ^2^Faculty of Medicine and Health Sciences, Universiti Malaysia Sarawak, 94300 Kota Samarahan, Sarawak, Malaysia

## Abstract

Various prevalence studies on* Leptospira* in animals and humans, as well as environmental samples, had been conducted worldwide, including Malaysia. However, limited studies have been documented on the presence of pathogenic, intermediate, and saprophytic* Leptospira* in selected animals and environments. This study was therefore conducted to detect* Leptospira* spp. in rats, soil, and water from urban areas of Sarawak using the polymerase chain reaction (PCR) method. A total of 107 rats, 292 soil samples, and 324 water samples were collected from April 2014 to February 2015. Pathogenic* Leptospira* was present in 5.6% (6/107) of rats, 11.6% (34/292) of soil samples, and 1.9% (6/324) of water samples. Intermediate* Leptospira* was present in 2.7% (8/292) of soil samples and 1.9% (6/324) of water samples. Saprophytic* Leptospira* was present in 10.3% (11/107) of rats, 1.4% (4/292) of soil samples, and 0.3% (1/324) of water samples. From this study, 76* Leptospira* spp. were isolated. Based on DNA sequencing, the dominant* Leptospira* spp. circulating in urban areas of Sarawak are pathogenic* Leptospira noguchii*, intermediate* Leptospira wolffii* serovar Khorat, and saprophytic* Leptospira meyeri*, respectively. Overall, this study provided important surveillance data on the prevalence of* Leptospira* spp. from rats and the environment, with dominant local serovars in urban areas of Sarawak.

## 1. Introduction

Leptospirosis is a zoonotic disease with worldwide distribution and caused by pathogenic* Leptospira*, which results in significant public health problem worldwide [1, 2]. The genus* Leptospira* consists of 20 species, with more than 300 serovars, grouped into 20 serogroups [3]. Based on the pathogenicity, they can be divided into three major clades, namely, pathogenic, saprophytic (nonpathogenic), and intermediate (unclear pathogenicity) [2]. The pathogenicity status of intermediate* Leptospira* remains a debate matter. For instance, hamsters inoculated with intermediate* L. inadai* and* L. licerasiae* do not cause any clinical manifestation of leptospirosis although both recovered from patients [4].

Humans usually get infected through direct contact with the infected animal urine via mucous membrane and exposed skin or indirect contact by exposure to the contaminated soil, water, and food [5–7]. Normally, maintenance hosts are asymptomatic while accidental hosts like humans may suffer a wide range of clinical manifestation such as renal failure, hepatic failure, severe pulmonary haemorrhage, and even death [8]. The overall case mortality rate in humans ranges from 1 to 5% and the elderly are claimed to have higher infection risk of leptospirosis [9]. Incidence rates are often underestimated because of the relative inaccessibility, lack of rapid diagnostics, and insufficient awareness of leptospirosis [10].

Similar prevalence studies on* Leptospira* spp. had been conducted in Malaysia in recent years.* Leptospira* had been isolated from urban rats of Kuala Lumpur [11] as well as soil and water from urban sites in Peninsular Malaysia [12]. The presence of* Leptospira* spp. was also reported in National Service Training Centres of Peninsular Malaysia [13]. Moreover, Thayaparan et al. [14] reported the presence of* Leptospira* spp. in wildlife around tourism areas in Sarawak. Our previous study highlighted the presence of* Leptospira* spp. in national parks [15]. The presence of* Leptospira* spp. in this area posed the risk of transmission and infection to humans. Since this study on the detection of* Leptospira* spp. in rats, soil and water from urban areas is first reported in Sarawak, the data obtained is definitely important to provide an insight on the status of* Leptospira* spp. in Sarawak.

Current diagnostic methods for* Leptospira* spp. usually rely on the demonstration of serum antibodies by microscopic agglutination test (MAT). However, this method is time-consuming, requires live strain of* Leptospira* in exponential culture phase, and gives subjective interpretation of result [16]. The use of PCR is therefore gaining more popularity over MAT now. PCR, as direct genome detection method, offers various key advantages such as reduced contamination, increased detection accuracy, faster detection, and elimination of the need to use many antigens [6]. Besides, the use of PCR is critical particularly at early stage of infection and acute stage of illness before antibodies are detectable. The early diagnosis of leptospirosis can reduce the complications due to leptospirosis and prevent fatality [17].

Many different primer sets had been applied in the detection of* Leptospira*. In this study,* LipL32*,* rRNA*, and* rrs* genes were targeted as reported in our previous study [15].* LipL32* gene is a major outer membrane lipoprotein of* Leptospira*. The lipoprotein is highly conserved among pathogenic* Leptospira* and considered as virulence factor where higher levels of* LipL32* are expressed in* Leptospira* during acute lethal infections than* Leptospira* cultured in vitro [8, 18].* rRNA* gene is very conserved throughout the bacterial kingdom. The primer set targeting* 16S rRNA* genes is genus-specific and useful in epidemiological study [19]. The primer set designed within the* rrs* gene encoding* 16S rRNA* is specific to saprophytic* Leptospira* [20].

The objective of this study was to determine the diversity of* Leptospira* spp. in rats, soil, and water samples from urban areas of Sarawak using PCR. This study also aimed to disseminate information on the dominant* Leptospira* serovars circulating in Sarawak so that control and preventive measures for this disease can be taken effectively.

## 2. Materials and Methods

### 2.1. Study Sites

This study focused on urban areas in Sarawak which are usually characterized by high density of humans population. According to Population and Housing Census 2010 by Department of Statistics Malaysia (2015), urban area is defined as gazetted areas with their adjoining built-up areas, which had a combined population of 10,000 or more at the time of the Census 2010 or the special development area that can be identified, which at least had a population of 10,000 with at least 60 % of population (aged 15 years and above) being involved in nonagricultural activities.

**Figure 1:**
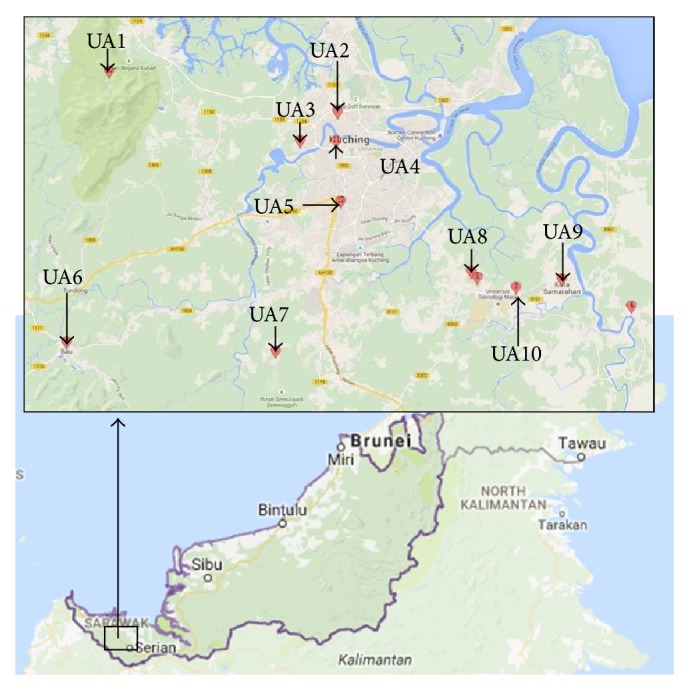
Location of all the urban areas (UA) examined in this study (MapCustomizer, 2016). UA1, UA2, UA3, and UA7 are densely populated villages; UA4 is a famous market near villages; UA5 is a densely populated residential area with prominent hawker centres; UA6 and UA9 are small villages; UA8 is a public university; and UA10 is a residential area.

The study sites (Figure 1) included public university, villages, residential areas, commercial centres, hawker centres, and markets. The selection of study sites was based on the recent reports of leptospirosis outbreak, environmental exposure due to humans activities, the suitability of the habitat for the rats breeding, and its likelihood of transmitting the disease. A total of 107 rats, 292 soil samples and 324 water samples were collected from April 2014 to February 2015.

In brief, the cage traps were set randomly near the garbage disposal areas, landfills, surrounding of housing areas, and store room. Soil samples were collected from the surrounding of housing areas, landfills, open field, and surrounding of lake. Water samples were collected from drain effluent, river, lake, and puddle.

### 2.2. Rat Trapping and Species Identification

The rats were caught alive using rectangular wire cage traps measuring 15 × 12.5 × 29 cm. Each trapping session took at least three consecutive days where the cage traps with baits were set up on the first day before checking for the capturing yield on the consecutive days. All the trapped rats under the family Muridae were euthanized humanely by placing them into a cloth bag containing cotton wool soaked with chloroform. They were further identified based on phenotypic characteristics such as fur colour (ventral and dorsal) and morphological measurements such as ear length, hind foot, tail length, and head body length [21].

### 2.3. Rat Samples Collection

Selected organs such as kidney and liver were removed from euthanized rats using sterile blade. A small piece of tissue was inoculated into modified semisolid Ellinghausen-McCullough-Johnson-Harris (EMJH) media with 100 *μ*g/mL 5-fluorouracil. All the enriched cultures were incubated aerobically at room temperature for 3 months. They were examined for the presence of* Leptospira* using polymerase chain reaction (PCR) every month [7, 11].

### 2.4. Soil and Water Samples Collection

Approximately 20 g soil and 50 mL water samples were collected. Soil samples were mixed vigorously using sterile distilled water and allowed to settle for 15 minutes prior to filtration using sterile 0.2 *μ*m pore size membrane filter (Sartorius AG, Germany). Water samples were filtered directly using the same type of membrane filter. About 1 mL of the samples was inoculated into modified semisolid EMJH media with 100 *μ*g/mL 5-fluorouracil. They were incubated aerobically at room temperature for 1 month [12, 13].

### 2.5. Molecular Detection of* Leptospira* spp. 

DNA extraction was carried out using Wizard™ Genomic DNA Purification Kit (Promega Corporation, USA) following the manufacturer's instructions prior to specific PCR amplification. Three sets of primers were used to target* LipL32*,* 16S rRNA*, and* rrs* genes in pathogenic, intermediate, and saprophytic* Leptospira*, respectively [15]. Pathogenic* Leptospira noguchii* strain LT796, intermediate* Leptospira wolffii* serovar Khorat strain Khorat-H2, and saprophytic* Leptospira meyeri* strain Sant-1 were used as positive controls.

The 25 *µ*L reaction mixtures included 5 *µ*L of 5x PCR buffer, 2.0 mM MgCl_2_, 0.4 *μ*M of each primer pair, 0.2 mM of dNTP mix, 1.25 U of* Taq* DNA polymerase (Promega Corporation, USA), and 5 *μ*L of DNA template. The cycling conditions included initial denaturation at 95°C for 2 min, 35 cycles of each of denaturation at 95°C for 1 min, primer annealing at 55°C for 30 sec, and extension at 72°C for 1 min, further extension at 72°C for 5 min, and indefinite holding period at 4°C. Electrophoresis was then carried out using 2% agarose gel in 1x TBE buffer.

### 2.6. Identification of* Leptospira* spp. 

At least one positive sample from each study site was chosen for DNA sequencing, depending on the availability of the sample sources. The amplicons from representative positive samples were subjected to commercial facility for sequencing (First BASE Laboratories Sdn Bhd, Malaysia). The sequencing data were compared with the GenBank database using nucleotide BLAST from National Center for Biotechnology Information (NCBI).

## 3. Results

**Figure 2:**
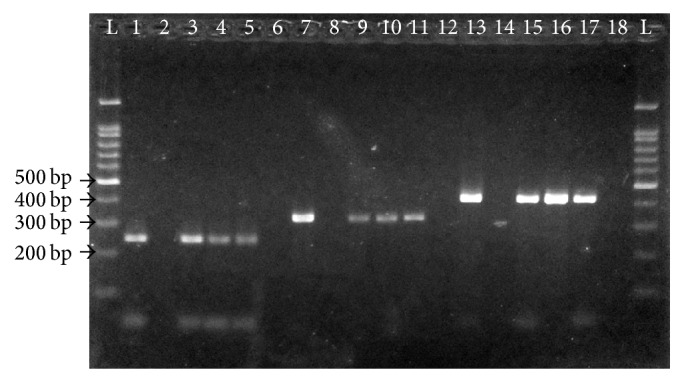
Representative gel image of PCR amplification. Lanes L, 100 bp DNA ladder; lanes 1, 7, and 13, positive controls; lanes 2, 8, and 14, negative controls; lanes 3 to 5, positive PCR amplicons specific to saprophytic* Leptospira* at 240 bp; lanes 9 to 11, positive PCR amplicons specific to intermediate* Leptospira* at 331 bp; lanes 15 to 17, positive PCR amplicons specific to pathogenic* Leptospira* at 423 bp; lanes 6, 12, and 18, negative PCR amplicons.

The agarose gel image for representative PCR amplicons in this study is shown in Figure 2. The prevalence of* Leptospira* spp. in rats, soil, and water samples is tabulated in Table 1. One hundred and seven rats comprising seven species were successfully collected. These rats consisted of 54.2% males and 75.7% adults. It was noticed that 87.9% of the total rats were* Rattus rattus*.

In this study, eleven female and twelve adult rats were* Leptospira* positive. Pathogenic* Leptospira* was present in six* Rattus rattus*, thirty-four soil samples, and six water samples. Eight soil samples and six water samples were detected with intermediate* Leptospira*. Saprophytic* Leptospira* was present in eleven rats, four soil samples, and one water sample.

Using PCR amplification, a total of 6.4% (46/723) pathogenic, 1.9% (14/723) intermediate, and 2.2% (16/723) saprophytic* Leptospira* were detected in rats, soil, and water samples from urban areas of Sarawak. The representative positive samples were made up of sixteen pathogenic* Leptospira* (four rats, eight soil samples, and four water samples), four intermediate* Leptospira* (one soil sample and three water samples), and ninesaprophytic* Leptospira* (six rats, two soil samples, and one water sample).

The nucleotide BLAST results for the representative positive samples were tabulated in Table 2. The degree of homology for pathogenic* Leptospira* varied from 75% to 91%. Based on phylogeny,* Leptospira interrogans* serovar Icterohaemorrhagiae and* Leptospira noguchii* are present dominantly in the rats. The dominant pathogenic* Leptospira* found in soil and water samples are* Leptospira noguchii* and* Leptospira interrogans* serovar Hardjo, respectively.

Positive samples showed 96% to 99% identity with reported sequences in intermediate* Leptospira*.* Leptospira wolffii* serovar Khorat is the dominant intermediate* Leptospira* in soil and water samples from urban areas. Apart from that, a high homology ranging from 95% to 99% was obtained for positive samples of saprophytic* Leptospira*.* Leptospira meyeri* is present in all the rats, soil, and water samples.

## 4. Discussion

In this study,* Rattus rattus* is the dominant rat species found in urban areas of Sarawak. This rat species is commonly known as black rat which adapts very well to the climatic and environmental conditions. In a study where 90 rodents were trapped in Cotonou, West Africa,* Rattus rattus* is the rat species with the greatest distribution as it spreads inland and is present in small towns and villages [7].* Rattus rattus* was also reported to be one of the principal domestic rats found in urban areas, open fields, and residential areas of Malaysia. Previous study on the distribution of urban rodents in Kuala Lumpur indicated that 91.8% (89/97) rodents trapped were* Rattus rattus* [22].

From the total 17* Leptospira* positive rats, a greater number of female (64.7%) and adult (70.6%) rats were detected. This is contrary with the finding by Mosallanejad et al. [23] who studied the seroprevalence of* Leptospira* in 120 wild rats in Iran. They reported that positive* Leptospira* was found in 2.5% male and 0.83% female rats. Nonetheless, our finding is in agreement with previous study by Benacer et al. [11] who stated that adult rats (7.2%) were infected more frequently than juvenile rats (5.4%). This is because adult rats are more aggressive during fighting which facilitates the transmission of* Leptospira* among them.

Pathogenic* Leptospira* was found in three rats captured and four water samples from UA8, a public university. Two water samples and one soil sample were contaminated by intermediate and saprophytic* Leptospira*, respectively. The presence of* Leptospira* in public university suggested that the occurrence of* Leptospira* may be related to humans settlement due to generation of significant amount of waste [24]. Indiscriminate disposal of food waste can lead to breeding of rats and other domestic animals.

In UA9, one of the small villages, saprophytic* Leptospira* was present in four rats captured whereas two soil samples and one water sample were contaminated by pathogenic* Leptospira*. These two contaminated soil samples were collected from surrounding of houses whereas contaminated water sample was collected from the river in this village. Many domesticated animals such as dogs, cats, and chickens can be found here. Since only environmental samples but no rats were detected with pathogenic* Leptospira*, it implied the possibility of domestic animals as the reservoir for* Leptospira*. This can result in the transmission of* Leptospira* through indirect contact with urine-contaminated environment [16]. Our finding is supported by Issazadeh et al. [25] who studied the distribution of* Leptospira* in surface waters among different region in Guilan Province, China. They reported a higher frequency of* Leptospira* in a region with more domestic animals such as cow, sheep, and horse. These animals are excellent reservoirs for* Leptospira*.

Sampling at a few densely populated villages (UA1, UA2, UA3, and UA7) and residential area (UA10) showed that three rats were positive towards pathogenic* Leptospira* and five rats were positive towards saprophytic* Leptospira*. Low prevalence of* Leptospira* spp. in residential area is consistent with the finding by Khairani-Bejo et al. [17] who reported that only 3.1% (1/32) rat captured in residential area of Serdang, Selangor was infected with* Leptospira*. Six soil samples collected from the surrounding of houses were contaminated by pathogenic* Leptospira*. One water sample contaminated with pathogenic* Leptospira* was collected from an artificial pond in residential area. From the observation during sampling, this artificial pond contains stagnant water which favours the survival of* Leptospira*. Higher concentration of* Leptospira* was found in stagnant river water than in rainwater or underground water [26].

Only eight soil samples and four water samples from a small village, UA6, were positive for intermediate* Leptospira*. These soil samples were collected from forest area whereas the water samples were collected from a small river in the village. There were nine soil samples with positive pathogenic* Leptospira* collected from landfills and surrounding of lake in commercial centres. Landfills with garbage containers have abundance of food resources which attract a lot of rats to forage. Rubbish can be found at the surrounding of lake. It was stipulated that the hygiene at this area is very poor. Improper waste management strongly encouraged the proliferation of* Leptospira* [12].

Five soil samples collected from UA5, a densely populated residential area with prominent hawker centres, were contaminated by pathogenic* Leptospira*. In another famous market, UA4, which comprised wet and dry markets, cafeteria, and food court, two rats captured carried saprophytic* Leptospira*. A total of 40.0% (12/30) soil samples were contaminated by pathogenic* Leptospira*. Besides, saprophytic* Leptospira* was present in three soil samples and one water sample in this market. Soil samples were mainly collected from open field in this large market whereas the water sample was collected from the river. The accumulation of garbage and food waste is a common scenario in market. This attracts rodents and domestic animals to populate and become potential reservoirs for* Leptospira*. From the observation during sampling, it was proven that the garbage and rubbish were disposed everywhere by public. Although the rats captured here did not carry pathogenic* Leptospira*, there is still a risk of infection to humans as the environment is highly contaminated with pathogenic* Leptospira*.

The presence of pathogenic* Leptospira* in rats was not surprised as the isolation of this spirochete from rats was first reported in 1917 [27]. The presence of* Leptospira* especially pathogenic strains in environmental soil and water is an index of leptospirosis in rats or domestic animals which have access to these sources [28]. Besides rats, other domestic animals may serve as reservoirs for* Leptospira* spp. and contaminate the environment in these study sites. The finding from previous researcher indicated that higher prevalence of* Leptospira* was detected in other animals such as cattle (53.8%), horse (27.9%), buffalo (58.7%), and donkey (40.0%) than in rats (3.3%) in Ahvaz, Iran. This is because those animals which live in groups near water may have additional access to the contaminated environment and stagnant water than rats [23].

The dominant pathogenic* Leptospira* serovar circulating in the urban areas is* Leptospira noguchii*.* L. noguchii* has been isolated from multiple animal species in Argentina, Barbados, and Nicaragua as well as from humans in the United States of America and neighbouring Panama and Peru [29]. Previous study by Silva et al. [30] also reported the isolation of three* Leptospira noguchii* strains from Brazil, of which two humans were infected by leptospirosis after contact with dogs and rats and one fatal leptospirosis case involving a dog. Nonetheless,* Leptospira* Icterohaemorrhagiae was the main serovar detected in the serum samples of rats captured in national service training centres in Kelantan and Terengganu [31]. This serovar is often associated with rodents especially rats worldwide [17]. Alexander et al. [32] also stated that serovar Icterohaemorrhagiae is the most common pathogenic strain found in Malaysian water.

Our result indicated that* Leptospira wolffii* serovar Khorat is the dominant intermediate* Leptospira* circulating in urban areas. It was previously isolated from wildlife and humans from Iran, India, Thailand, and Sabah [33]. Benacer et al. [12] isolated* Leptospira wolffii* from University Malaya Lake in Peninsular Malaysia. The presence of intermediate species such as* Leptospira wolffii* in clinical humans samples and animals indicated the circulation of this strain which may play a role in the transmission cycle within animal reservoir and humans [2]. On the other hand,* Leptospira meyeri* is found to be dominant among saprophytic* Leptospira* in urban areas. The same strain had been isolated from the soil at Pantai Dalam, Kuala Lumpur [12].

Different* Leptospira* serovars may circulate in different reservoirs or environment in a particular geographical region [2]. The knowledge of locally circulating serovars and their reservoirs is important to the epidemiological understanding of this disease in a region [34]. Dominant serovars can be changed in a region from time to time. This is because old and well-established serovars may adapt themselves into new hosts, which subsequently create risk of infection [35, 36].

As a whole, this study provided an insight on the diversity of* Leptospira* spp. in urban areas of Sarawak, Malaysia. Various precaution measures such as rodent control campaign should be taken to reduce the risk of leptospirosis to humans.

## Figures and Tables

**Table 1:** Prevalence of *Leptospira* spp. by PCR in rats, soil, and water samples collected in this study.

Sample	Pathogenic			Intermediate			Saprophytic	
	Number.^a^	%^b^		Number.^a^	%^b^		Number.^a^	%^b^
Rat	6/107	5.6		0/107	0.0		11/107	10.3
Soil	34/292	11.6		8/292	2.7		4/292	1.4
Water	6/324	1.9		6/324	1.9		1/324	0.3

Note: _ _^a^number of positive samples/number of samples collected; _ _^b^prevalence (in %) of positive samples among the samples collected.

**Table 2:** Nucleotide BLAST results showing similarity with *LipL32* gene of pathogenic *Leptospira*, *16S rRNA* gene of intermediate *Leptospira*,and *rrs* gene of saprophytic *Leptospira* from GenBank.

Sample ID	Sample	Accession number	Description	Maximum score	Total score	Query coverage	Maximum identity
*Pathogenic*							
CFP4	Rat	KC800993.1	*Leptospira interrogans *serovar Icterohaemorrhagiae strain RTCC 2812 outer membrane protein (lipL32) gene, partial cds	525	525	99%	89%
CFP5	Rat	KC800993.1	*Leptospira interrogans *serovar Icterohaemorrhagiae strain RTCC 2812 outer membrane protein (lipL32) gene, partial cds	486	486	99%	87%
CFP39	Rat	AY461920.1	*Leptospira noguchii *strain LT796 LipL32 (lipL32) gene, partial cds	512	512	97%	88%
CFP40	Rat	AY461920.1	*Leptospira noguchii *strain LT796 LipL32 (lipL32) gene, partial cds	486	486	94%	90%
CFP24	Soil	KF297610.1	*Leptospira weilii *clone lipL32-122069 LipL32 gene, partial cds	462	462	72%	91%
CFP25	Soil	AY461920.1	*Leptospira noguchii *strain LT796 LipL32 (lipL32) gene, partial cds	510	510	94%	88%
CFP29	Soil	AY461924.1	*Leptospira noguchii *strain LT796 LipL32 (lipL32) gene, partial cds	286	286	99%	75%
CFP32	Soil	AY461920.1	*Leptospira noguchii *strain LT796 LipL32 (lipL32) gene, partial cds	521	521	97%	89%
CFP37	Soil	AY461920.1	*Leptospira noguchii *strain LT796 LipL32 (lipL32) gene, partial cds	542	542	94%	90%
CFP34	Soil	AY609333.1	*Leptospira borgpetersenii *serovar Mini major outer membrane protein (lipl32) gene, complete cds	508	508	93%	88%
CFP35	Soil	AY609333.1	*Leptospira borgpetersenii *serovar Mini major outer membrane protein (lipl32) gene, complete cds	518	518	98%	89%
CFP36	Soil	AY461920.1	*Leptospira noguchii *strain LT796 LipL32 (lipL32) gene, partial cds	514	514	97%	88%
CFP7	Water	AY609333.1	*Leptospira borgpetersenii *serovar Mini major outer membrane protein (lipl32) gene, complete cds	510	510	99%	88%
CFP9	Water	JN886739.1	*Leptospira interrogans *serovar Hardjo strain RTCC2821 Lipl32 (lipl32) gene, partial cds	494	494	99%	88%
CFP28	Water	JN886739.1	*Leptospira interrogans *serovar Hardjo strain RTCC2821 Lipl32 (lipl32) gene, partial cds	336	336	99%	77%
CFP31	Water	CP006723.1	*Leptospira interrogans *serovar Linhai str. 56609 chromosome 1, complete sequence	525	525	93%	89%

*Intermediate*							
CFG16	Soil	NR_044042.1	*Leptospira wolffii *serovar Khorat strain Khorat-H2 16S ribosomal RNA gene, partial sequence	562	562	99%	97%
CFG7	Water	NR_044042.1	*Leptospira wolffii *serovar Khorat strain Khorat-H2 16S ribosomal RNA gene, partial sequence	592	592	97%	99%
CFG17	Water	AY631891.1	*Leptospira inadai *serovar Aguaruna strain MW 4 16S ribosomal RNA gene, partial sequence	545	545	97%	96%
CFG19	Water	NR_044042.1	*Leptospira wolffii *serovar Khorat strain Khorat-H2 16S ribosomal RNA gene, partial sequence	584	584	98%	99%

*Saprophytic*							
CFS15	Rat	KP739778.1	*Leptospira meyeri *strain 19CAP 16S ribosomal RNA gene, partial sequence	431	431	98%	98%
CFS16	Rat	KP739778.1	*Leptospira meyeri *strain 19CAP 16S ribosomal RNA gene, partial sequence	425	425	98%	98%
CFS17	Rat	KP739778.1	*Leptospira meyeri *strain 19CAP 16S ribosomal RNA gene, partial sequence	388	388	96%	95%
CFS18	Rat	KP739778.1	*Leptospira meyeri *strain 19CAP 16S ribosomal RNA gene, partial sequence	353	353	91%	99%
CFS19	Rat	KP739778.1	*Leptospira meyeri *strain 19CAP 16S ribosomal RNA gene, partial sequence	446	446	99%	99%
CFS36	Rat	KP739778.1	*Leptospira meyeri *strain 19CAP 16S ribosomal RNA gene, partial sequence	416	416	98%	97%
CFS4	Soil	JQ988852.1	*Leptospira meyeri *strain Semaranga_DB49 16S ribosomal RNA gene, partial sequence	433	433	98%	98%
CFS21	Soil	KP739778.1	*Leptospira meyeri *strain 19CAP 16S ribosomal RNA gene, partial sequence	418	418	98%	97%
CFS20	Water	KP739778.1	*Leptospira meyeri *strain 19CAP 16S ribosomal RNA gene, partial sequence	412	412	98%	96%
